# Combined *Lactiplantibacillus plantarum* CRL1506 and MPL16 Nasal Priming More Effectively Modulates Respiratory Antiviral Innate Immunity than Single Strains

**DOI:** 10.3390/ijms262010079

**Published:** 2025-10-16

**Authors:** Luciano Arellano-Arriagada, Leonardo Albarracin, Kohtaro Fukuyama, Solange Cisterna-Vergara, Weichen Gong, Fu Namai, Keita Nishiyama, Yoshihito Suda, Haruki Kitazawa, Julio Villena

**Affiliations:** 1 Food and Feed Immunology Group, Laboratory of Animal Food Function, Graduate School of Agricultural Science, Tohoku University, Sendai 980-8572, Japan; 2 Laboratory of Respiratory Immunology (LaRI), Division of Animal Immunology and Omics, International Education and Research Center for Food and Agricultural Immunology (CFAI), Graduate School of Agricultural Science, Tohoku University, Sendai 980-8572, Japan; 3 Livestock Immunology Unit, International Education and Research Centre for Food and Agricultural Immunology (CFAI), Graduate School of Agricultural Science, Tohoku University, Sendai 980-8572, Japan; 4 Department of Food, Agriculture and Environment, Miyagi University, Sendai 980-8572, Japan

**Keywords:** immunobiotic lactobacilli, TLR3, RSV, PRRSV, alveolar macrophages

## Abstract

This study evaluated whether the combined *Lactiplantibacillus plantarum* CRL1506 and MPL16 nasal priming more effectively modulated the Toll-like receptor (TLR)-3- and respiratory syncytial virus (RSV)-mediated respiratory immune responses in mice than single strains. The interaction of single and combined strains with porcine alveolar macrophages (AMs) and porcine respiratory epithelial cells (PBE cells) in the context of TLR3 activation and porcine reproductive and respiratory syndrome virus (PRRSV) was also evaluated. The in vivo studies in mice revealed that the CRL1506 + MPL16 combination was more effective than the individual strains at reducing RSV replication, protecting the lung from TLR3-mediated inflammatory injury and modulating innate antiviral responses, particularly in AMs. In vitro, lactobacilli treatment also increased the resistance of porcine AMs to PRRSV infection. Notably, the CRL1506 + MPL16 combination was not more effective than the single strains in modulating AMs antiviral immunity. Complementary assays in PBE cells revealed that *L. plantarum* CRL1506 induced higher production of IFN-λ than the MPL16 strain in response to TLR3 activation. Thus, the superior in vivo protection against RSV seen with the *L. plantarum* CRL1506–MPL16 combination likely reflects complementary actions of lactobacilli: MPL16 would efficiently modulate AMs, whereas CRL1506 would be more effective to target respiratory epithelial cells driving greater IFN-λ production that further boosts AM antiviral activity. The results from the animal models of this work furnish the scientific basis for proposing future human trials to assess the efficacy of the CRL1506 + MPL16 combination in improving respiratory antiviral immunity.

## 1. Introduction

Acute respiratory infections are a global health problem associated with high morbidity and mortality particularly in high risk populations including children younger than 5 years and adults aged 70 years and older [1]. It has been informed that in 2021, these infectious diseases resulted in 19,600 deaths associated with upper respiratory infections [2] and more than 2 million deaths caused by infections in the lower respiratory tract [3]. It was also reported that *Streptococcus pneumoniae* is the most common cause of respiratory infection-associated deaths among bacteria, while influenza virus (IFV) and respiratory syncytial virus (RSV) are the leading pathogens among viruses [1,2,3]. As antimicrobial resistance escalates and the development of efficient and economical vaccines progresses slowly, the respiratory infections burden remains high. Therefore, it is still necessary to develop alternatives that replace or complement the tools currently available to prevent or treat respiratory infections.

In the last decade, it was shown that healthy infants had higher nasopharyngeal abundance of lactobacilli than individuals with RSV-associated acute respiratory infections [4]. In addition, higher abundance of lactobacilli were described in healthy infants’ respiratory tract who had a diminished risk of developing RSV-associated childhood wheezing [5]. Thus, these data show that certain lactobacilli are well adapted to the upper respiratory tract and may serve as beneficial keystone species in this niche [6]. In line with the human data, we and others have shown that the nasal priming with immunomodulatory lactobacilli, referred to us immunobiotics, can beneficially modulate immunity in the respiratory tract, particularly in the context of infectious diseases [7,8,9,10]. Several studies in mice have demonstrated that nasally administered immunomodulatory lactobacilli augmented resistance to viral infections, including those caused by IFV [11], RSV [7,8], and pneumonia virus of mice (PVM) [9,10] through the modulation of innate immunity. We found that prophylactic intranasal priming with *Lactiplantibacillus plantarum* (*L. plantarum*) CRL1506 recalibrated the respiratory innate immune response to either the Toll-like receptor (TLR)-3 agonist polyinosinic:polycytidylic acid [poly(I:C)] or RSV, resulting in better viral clearance and diminished inflammatory lung pathology [7]. Furthermore, in poly(I:C)-stimulated human respiratory Calu-3 cells, the immunobiotic *L. plantarum* strains MPL16 and CRL1506 modulated the production of interferon (IFN)-β, interleukin (IL)-6, and chemokines, as well as the expression of the antiviral factors DExD/H-box helicase 58 (DDX58), myxovirus resistance protein 1 (Mx1), and 2′-5′-oligoadenylate synthetase 1 (OAS1). Both strains also increased resistance to severe acute respiratory syndrome coronavirus 2 (SARS-CoV-2) challenge [12]. These results show the potential of the MPL16 and CRL1506 strains to be used as nasally administered immunobiotics to prevent or reduce the severity of viral infections in humans.

As the probiotic discipline advances and more beneficial strains are characterized, new questions have arisen such as whether single-strains are more effective than multi-strain mixtures to exert the probiotic effects. In this sense, it was suggested that the beneficial effects of single versus multi-strains depend not only on the specific bacteria but also on the specific conditions/diseases [13]. For instance, it was shown that the single administration of *Lacticaseibacillus rhamnosus* (*L. rhamnosus*) GG was significantly more protective for necrotizing enterocolitis than the mixture of this strain with *Bifidobacterium lactis* (*B. lactis*) Bb12. In contrast, the coadministration of *L. rhamnosus* GG with *B. lactis* Bb12 achieved significantly greater efficacy for the eradication of *Helicobacter pylori* (*H. pylori*) than the GG strain alone reviewed in [13]. In a study performed in mice infected with *H. pylori*, it was shown that the single administration of *Lacticaseibacillus rhamnosus* JB3, *Limosilactobacillus fermentum* P2, and *Lacticaseibacillus casei* L21 was as efficient as the combination of the three strains to decrease the pathogen loads in the stomach and to modulate the serum levels of IFN-γ and IL-1β [14]. However, the multistrain treatment was more effective to modulate the oxidative stress than single strains.

Most of the studies comparatively evaluating single versus multi strains probiotics have focused on the prevention of antibiotic-associated diarrhea and the eradication of *H. pylori*, while the prevention of respiratory infections was explored in a lesser extent [13]. It was shown in human clinical trials that the oral administration of *B. lactis* Bb12 was able to improve the outcome of respiratory infections while *L. rhamnosus* GG alone did not achieve this beneficial effect. In addition, the combination of the Bb12 and GG strains together did not show an increase in efficacy compared to the Bb12 strain alone despite being given for longer times [15,16]. To the best of our knowledge, there are no reports of studies comparing the impact of individual strains and the respective mixtures of probiotics, administered nasally, on the resistance to respiratory viral infections.

Considering this background, the aim of this study was to evaluate whether the nasal priming with combined *L. plantarum* CRL1506 and MPL16 was more effective than the single-strain administration to modulate the TLR3- and RSV-mediated respiratory immune responses in mice. We also evaluated the interaction of single and combined strains with porcine alveolar macrophages (AMs) and respiratory epithelial cells (PBE cells) in the context of TLR3 activation and porcine reproductive and respiratory syndrome virus (PRRSV) to evaluate the specific role of these cells in the beneficial effects of the immunobiotic strains. Defining whether single or multi strain probiotic formulations are optimal for the regulation of respiratory tract immunity and investigating the specific mechanisms underlying the beneficial effects of these strains could improve the development of lactobacilli-based biotechnological tools that can help to combat respiratory viral infections.

## 2. Results

### 2.1. Nasal Administration of Lactiplantibacillus plantarum Strains Improve TLR3-Mediated Respiratory Innate Antiviral Immune Response in Mice

**Figure 1:**
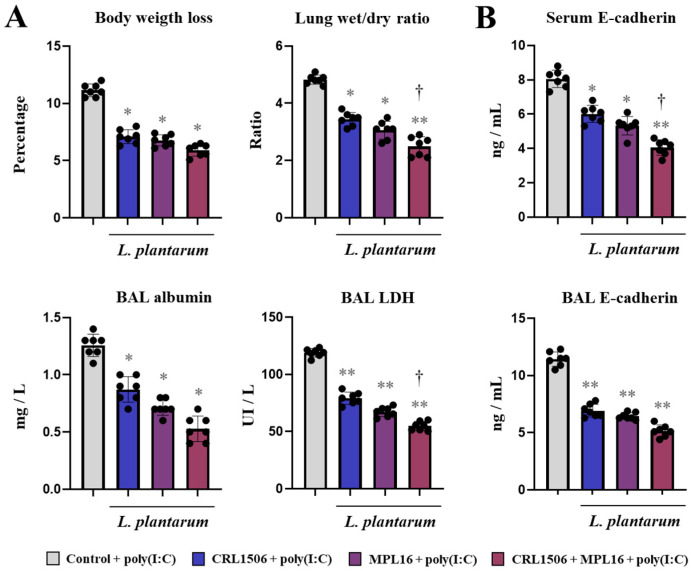
The effect of single and combined nasal administration of *Lactiplantibacillus plantarum* CRL1506 and MPL16 on the lung injuries induced by TLR3 activation. Mice were nasally treated with CRL1506, MPL16 or CRL1506 + MPL16 strains for 5 days and then stimulated with poly(I:C) on days 7, 8, and 9 via the nasal route. Two days after the last poly(I:C) administration the body weight loss, lung wet:dry ratio, the concentration of broncho-alveolar lavage (BAL) albumin, the activity of BAL LDH (**A**), and the concentrations of E-cadherin in BAL and serum (**B**) were determined. Mice not treated with lactobacilli and stimulated with poly(I:C) were used as controls. The results are shown as mean ± SD. Significant differences are shown compared to the control group at *p* < 0.05 (*) or *p* < 0.01 (**). Significant differences are shown compared to the single strain administration at *p* < 0.05 (†).

We first investigated whether the single or combined administration of *L. plantarum* CRL1506 and MPL16 could influence the TLR3-mediated immune response in the respiratory tract of mice (Figure 1). Then, animals were nasally primed with CRL1506, MPL16 or CRL1506 + MPL16 and subsequently stimulated with poly(I:C). As we reported previously [7,8], the activation of TLR3 in the respiratory tract induced injuries of lung tissue as demonstrated by the increase in body weight loss, lung wet/dry ratio (marker of oedema), and the levels of albumin (marker of altered alveolar capillary barrier) and lactate dehydrogenase (LDH) (marker of cytotoxicity) in broncho-alveolar lavage (BAL) samples. Of note, the three treatments with lactobacilli significantly reduced the levels of the lung injury markers (Figure 1A). The CRL1506, MPL16 or CRL1506 + MPL16 treatments were equally effective in reducing body weight loss and lung wet/dry ratio compared to controls but the combination of the strains was more efficient than single strain to reduce BAL albumin and LDH. It was shown that soluble E-cadherin can be measured in the serum and BAL fluid of mice as markers of the disruption of the airway epithelial barrier after the infection with RSV [17] or IFV [18]. We also evaluated the levels of serum and BAL E-cadherin after poly(I:C) stimulation. As shown in Figure 1B, there was a significant increase of E-cadherin in both serum and BAL after TLR3 activation in all the experimental groups. However, mice treated with lactobacilli had significantly lower values of both parameters compared to the controls. In addition, it was observed that mice treated with CRL1506 + MPL16 had lower serum E-cadherin levels than animals receiving single strains (Figure 1B).

**Figure 2:**
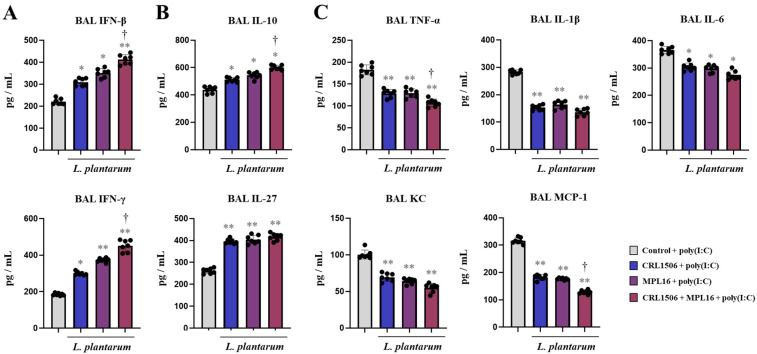
The effect of single and combined nasal administration of *Lactiplantibacillus plantarum* CRL1506 and MPL16 on the respiratory cytokine profile induced by TLR3 activation. Mice were nasally treated with CRL1506, MPL16 or CRL1506 + MPL16 strains for 5 days and then stimulated with poly(I:C) on days 7, 8, and 9 via the nasal route. Two days after the last poly(I:C) administration the concentrations of interferons (**A**), regulatory cytokines (**B**) and proinflammatory cytokines and chemokines (**C**) were determined. Mice not treated with lactobacilli and stimulated with poly(I:C) were used as controls. The results are shown as mean ± SD. Significant differences are shown compared to the control group at *p* < 0.05 (*) or *p* < 0.01 (**). Significant differences are shown compared to the single strain administration at *p* < 0.05 (†).

We next evaluated whether lactobacilli were capable of inducing a distinct cytokine profile in the respiratory tract after the stimulation with the TLR3 agonist poly(I:C). As we reported previously [7,8], the nasal administration of poly(I:C) to mice induced an increase in IFNs (Figure 2A), regulatory cytokines (Figure 2B) and inflammatory cytokines and chemokines (Figure 2C). The three lactobacilli treatments differentially modulated the respiratory cytokine profile inducing increases in IFN-β, IFN-γ (Figure 2A), IL-10, and IL-27 (Figure 2B) as well as reductions in tumor necrosis factor (TNF)-α, IL-1β, IL-6, keratinocyte-derived chemokine (KC) and monocyte chemoattractant protein-1 (MCP-1) (Figure 2B) in the BAL samples compared to the control group. Of note, the combination of the two lactobacilli was more efficient than single CRL1506 or MPL16 strains to modulate BAL IFN-β, IFN-γ, IL-10 and MCP-1.

### 2.2. Nasal Administration of Lactiplantibacillus plantarum Strains Improve Immunity Against RSV Infection in Mice

**Figure 3:**
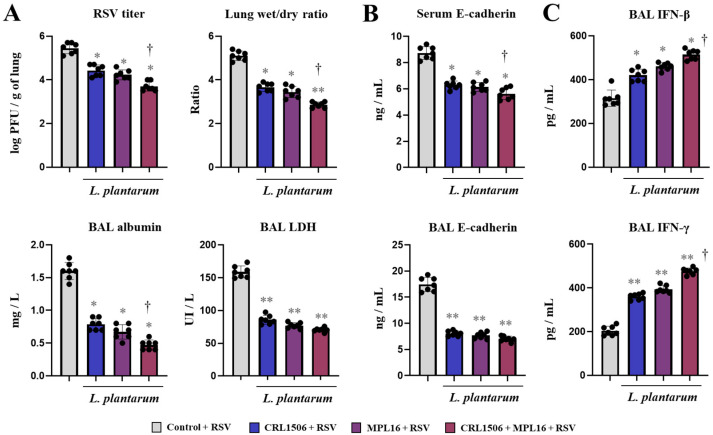
The effect of single and combined nasal administration of *Lactiplantibacillus plantarum* CRL1506 and MPL16 on the resistance to Respiratory Syncytial Virus (RSV) infection. Mice were nasally treated with CRL1506, MPL16 or CRL1506 + MPL16 strains for 5 days and then infected with RSV on day 7 via the nasal route. Two days after the viral infection the RSV titer, lung wet:dry ratio, the concentration of broncho-alveolar lavage (BAL) albumin, the activity of BAL LDH (**A**), the concentrations of E-cadherin in BAL and serum (**B**) and the levels of interferons in BAL (**C**) were determined. Mice not treated with lactobacilli and infected with RSV were used as controls. The results are shown as mean ± SD. Significant differences are shown compared to the control group at *p* < 0.05 (*) or *p* < 0.01 (**). Significant differences are shown compared to the single strain administration at *p* < 0.05 (†).

Considering the ability of the lactobacilli treatments to beneficially modulate the TLR3-mediated innate antiviral immune response in the respiratory tract, we next aimed to evaluate whether they can influence the immune response to a real viral challenge. Therefore, mice were nasally primed with CRL1506, MPL16 or CRL1506 + MPL16 and then infected with RSV, a negative-sense, single-stranded RNA virus from the Pneumoviridae family that can cause severe respiratory infections in children [1,2,3] and can efficiently infect mice [7,8]. The virus was detected in the lungs of mice after 48 h post-infection and it induced alterations of lung wet:dry ratio and BAL albumin and LDH levels (Figure 3A), in line with our previous publications [7,8]. We also detected increased levels of serum and BAL E-cadherin levels in mice infected with RSV (Figure 3B). Interestingly, mice treated with CRL1506, MPL16 or CRL1506 + MPL16 had significantly lower RSV titers and values of all the lung injury markers evaluated. Furthermore, it was observed that the CRL1506 + MPL16 treatment was more effective than the single-strain administration to reduce RSV titers as well as lung wet:dry ratio, BAL albumin (Figure 3A) and serum E-cadherin (Figure 3B). The improved ability of lactobacilli-treated mice to reduce RSV replication could be associated with their higher production of IFN-β and IFN-γ in the respiratory tract after the viral challenge (Figure 3C). It was also observed that the combination of *L. plantarum* CRL1506 and MPL16 treatment was more efficient than the single-strain administration to enhance BAL IFN-β and IFN-γ.

**Figure 4:**
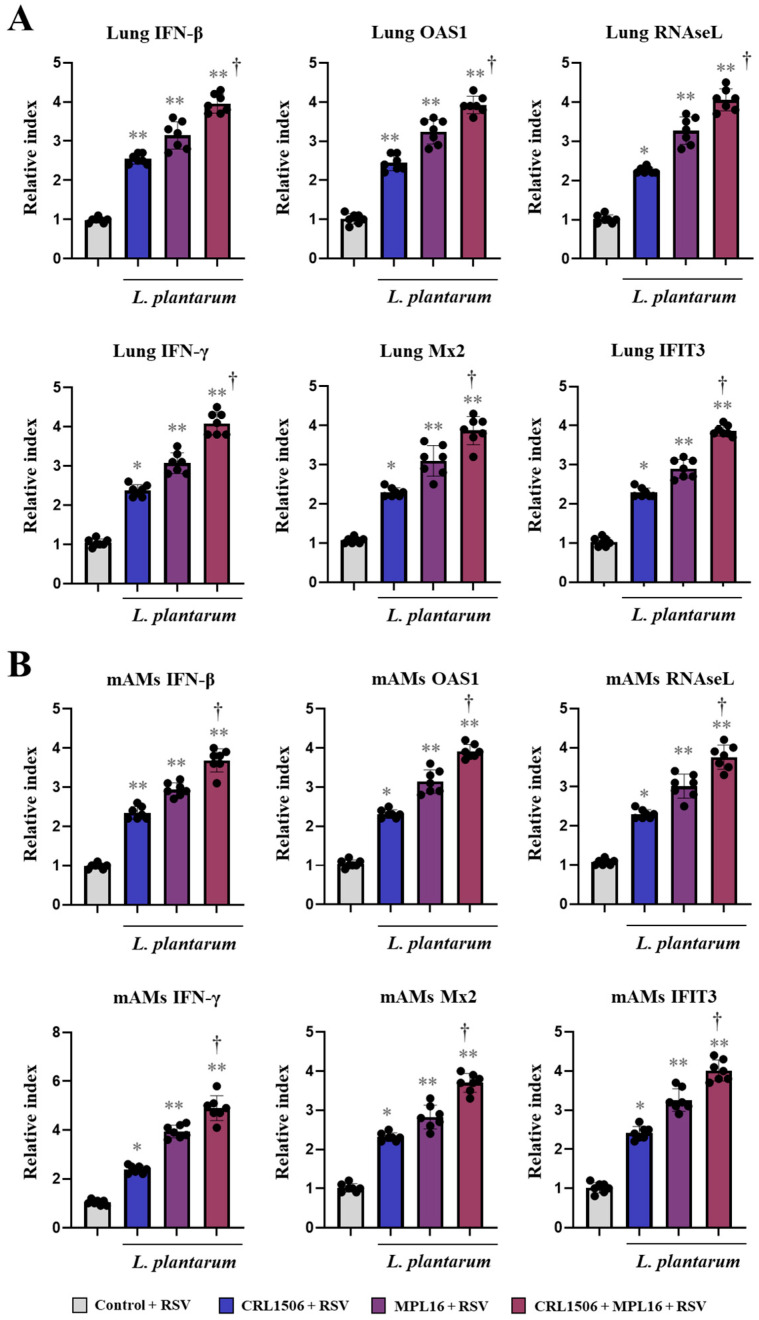
The effect of single and combined nasal administration of *Lactiplantibacillus plantarum* CRL1506 and MPL16 on the antiviral immune response induced by Respiratory Syncytial Virus (RSV) infection. Mice were nasally treated with CRL1506, MPL16 or CRL1506 + MPL16 strains for 5 days and then infected with RSV on day 7 via the nasal route. Two days after the viral infection the expression of interferon and antiviral factors genes were determined in the lung tissue (**A**), and in murine alveolar macrophages (mAMs) (**B**). Mice not treated with lactobacilli and infected with RSV were used as controls. The results are shown as mean ± SD. Significant differences are shown compared to the control group at *p* < 0.05 (*) or *p* < 0.01 (**). Significant differences are shown compared to the single strain administration at *p* < 0.05 (†).

In order to demonstrate that lactobacilli improved antiviral mechanisms through the activation of the expression of IFNs and IFN-induced genes, we measured the expressions of these factors in the lungs of RSV-infected mice (Figure 4A) as well as in alveolar macrophages (mAMs) (Figure 4B). As expected, the three lactobacilli treatments upregulated the expressions of *IFN-β* and *IFN-γ* in the lungs compared to the control group. In addition, lactobacilli-treated mice had significantly higher expression levels of lung *OAS1*, *Mx2*, *RNAseL*, and *IFIT3* than controls (Figure 4A). Similarly, mice that received CRL1506, MPL16 or CRL1506 + MPL16 treatments had improved expression of all the genes evaluated in mAMs (Figure 4B). Of note, it was observed that mice treated with CRL1506 + MPL16 had higher expressions of *IFN-β*, *IFN-γ*, *OAS1*, *Mx2*, *RNAseL*, and *IFIT3* in both the lungs (Figure 4A) and mAMs (Figure 4A) compared to animals receiving single strains.

### 2.3. Lactiplantibacillus Plantarum Strains Improve TLR3-Mediated Innate Antiviral Immune Response in Porcine Alveolar Macrophages

The experiments performed with mice indicated that CRL1506, MPL16 and CRL1506 + MPL16 would be able to modulate the ability of AMs to respond to TLR3 activation or respiratory viral infection. Then, we aimed to evaluate the capacity of the three lactobacilli treatments to modulate the antiviral immune response specifically in AMs. For this purpose, we selected the porcine cell line PAM-KNU (pAMs) and a virus that is able to replicate in pAMs (PRRSV) for further studies.

**Figure 5:**
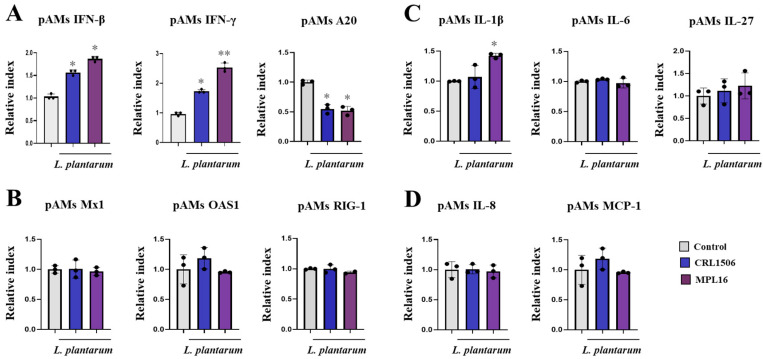
The effect of *Lactiplantibacillus plantarum* CRL1506 and MPL16 on the cytokine profile of porcine alveolar macrophages. Porcine alveolar macrophages (pAMs) were stimulated with CRL1506 or MPL16 strains for 24 h and the expressions of interferons (**A**), antiviral factors (**B**), cytokines (**C**) and chemokines (**D**) genes were determined. pAMs not treated with lactobacilli used as controls. The results are shown as mean ± SD. Significant differences are shown compared to the control group at *p* < 0.05 (*) or *p* < 0.01 (**).

In the first set of experiments, pAMs were stimulated with single CRL1506 or MPL16 strains and the expressions of IFNs (Figure 5A), antiviral factors (Figure 5B), cytokines (Figure 5C) and chemokines (Figure 5D) were evaluated. Both strains increased the expressions of *IFN-β* and *IFN-γ* and reduced *A20* (Figure 5A) in basal non-inflammatory conditions. In addition, *L. plantarum* MPL16 increased *IL-1β* (Figure 5C). No differences were found between lactobacilli-treated pAMs and controls when the expressions of *Mx1*, *OAS1, RIG-1, IL-6, IL-27*, *IL-8* or *MCP-1* genes were compared.

**Figure 6:**
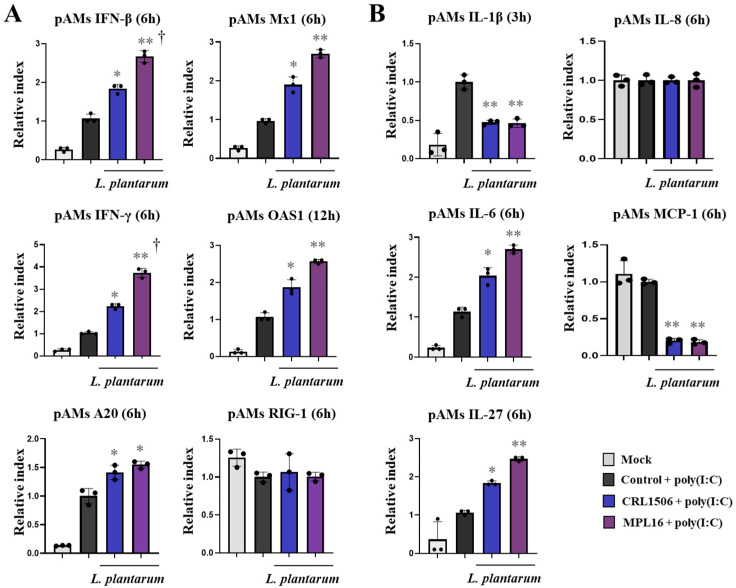
The effect of *Lactiplantibacillus plantarum* CRL1506 and MPL16 on the cytokine profile of porcine alveolar macrophages induced by TLR3 activation. Porcine alveolar macrophages (pAMs) were stimulated with CRL1506 or MPL16 strains for 24 h and then stimulated with poly(I:C) for 3, 6 or 12 h. The expressions of interferons, antiviral factors, the regulatory factor A20 (**A**), and proinflammatory and regulatory cytokines (**B**) genes were determined. pAMs not treated with lactobacilli and stimulated with poly(I:C) were used as controls. The expressions of immune factors in pAMs without any stimuli (mock) are shown. The results are shown as mean ± SD. Significant differences are shown compared to the poly(I:C)-stimulated control group at *p* < 0.05 (*) or *p* < 0.01 (**). Significant differences are shown compared to the CRL1506 strain administration at *p* < 0.05 (†).

In the second set of experiments, pAMs were stimulated with lactobacilli, challenged with poly(I:C) and the expression of IFNs, antiviral factors, cytokines and chemokines were evaluated after 3, 6 and 12 h (Figure 6 and Supplementary Figures S1 and S2). The stimulation of control pAMs with the TLR3 agonist significantly increased the expressions of *IFN-β* from h 3 post-stimulation while *IFN-γ* was enhanced only at h 6 compared with non-stimulated cells. In addition, it was detected that *Mx1* was augmented from h 6, *OAS1* was increased from h 12 and *RIG-1* was enhanced only at h 6 in control pAMs compared to non-stimulated cells (Figure 6A, Supplementary Figure S1). The negative regulator of the TLR signaling *A20* was increased in pAMs at h 3, 6 and 12 compared to basal conditions. Of note, lactobacilli-treated pAMs showed increased expression levels of *IFN-β* (3 h), *IFN-γ* (3, 6 and 12 h), *Mx1* (6 h), *OAS1* (12 h) and *A20* (6 h) than controls (Figure 6A). In addition, both CRL1506 and MPL16 treatments reduced *IFN-β* expression at h 12 (Supplementary Figure S1). *L. plantarum* MPL16 was more efficient than the CRL1506 strain to modulate *IFN-β* and *IFN-γ* expression in pAMs (Figure 6A).

Surprisingly, the challenge of pAMs with poly(I:C) did not induce effects on *IL-8* expression in none of the experimental groups while *IL-1β* (3, 6 h), *IL-6* (3, 6 h), and *MCP-1* (3 h) genes were significantly enhanced compared to non-stimulated cells (Figure 6B, Supplementary Figure S2). In addition, no differences were observed between poly(I:C)-challenged and non-stimulated pAMs when *IL-27* expression was evaluated. Interestingly, CRL1506 and MPL16 significantly decreased and increased *IL-1β* expression at h 3 and 12, respectively. Both strains also augmented *IL-6* and *IL-27* expressions and reduced *MCP-1* at h 6 (Figure 6B). Only *L. plantarum* MPL16 increased *MCP-1* and *IL-27* at h 12 (Supplementary Figure S2).

### 2.4. Lactiplantibacillus Plantarum Strains Improve Immunity Against PRRSV in Porcine Alveolar Macrophages

**Figure 7:**
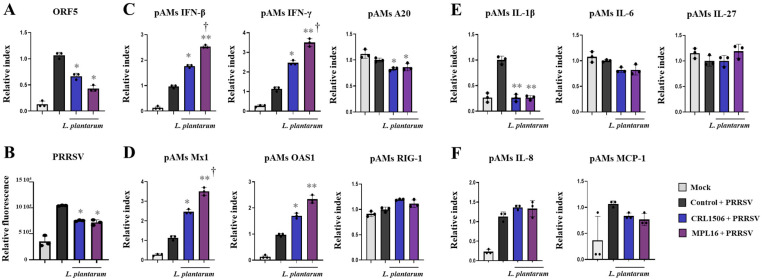
The effect of *Lactiplantibacillus plantarum* CRL1506 and MPL16 on the immune response of porcine alveolar macrophages to Porcine Reproductive Respiratory Syndrome Virus (PRRSV) infection. Porcine alveolar macrophages (pAMs) were stimulated with CRL1506 or MPL16 strains for 24 h and then infected with PRRSV for 24 h. The viral replication (**A**,**B**) and the expressions of interferons, the regulatory factor A20 (**C**), antiviral factors (**D**), cytokines (**E**) and chemokines (**F**) genes were determined. pAMs not treated with lactobacilli and infected with PRRSV were used as controls. The expressions of immune factors in pAMs without any stimuli (mock) are shown. The results are shown as mean ± SD. Significant differences are shown compared to the PRRSV-infected control group at *p* < 0.05 (*) or *p* < 0.01 (**). Significant differences are shown compared to the CRL1506 strain administration at *p* < 0.05 (†).

We next investigated whether *L. plantarum* CRL1506 or MPL16 could improve the resistance of pAMs to a real viral challenge. For this purpose, we selected PRRSV, a positive-sense, single-stranded RNA virus of the Arteriviridae family that causes reproductive failure in sows and respiratory disease in pigs, and efficiently infects porcine macrophages. Then, we designed experiments to challenge pAMs with PRRSV after the treatment with lactobacilli. Results showed that pAMs were efficiently infected with PRRSV as shown by the determination of the viral gene *ORF5* (open reading frame 5) by RT-qPCR (Figure 7A) and the viral antigens by IF (Figure 7, Supplementary Figure S3). The study of the expression of IFNs, antiviral factors, cytokines and chemokines after 6 (Supplementary Figure S4) and 24 (Figure 7C–F) h pots-infection with PRRSV showed significant increases in *IFN-β*, *IFN-γ*, *Mx1*, and *OAS1* only at h 24 after the viral challenge, compared to non-infected pAMs. In control infected pAMs, no changes in the expressions of *RIG-1* or *A20* were detected in comparison to non-infected cells both at 6 (Supplementary Figure S4A,B) and 24 (Figure 7A,B) h post-infection. It was also observed that CRL1506 and MPL16 strains enhanced the expressions of *IFN-β* (24 h), *IFN-γ* (6, 24 h), *Mx1* (24 h), and *OAS1* (6, 24 h) and reduced *A20* (6, 24 h) in PRRSV-infected pAMs. *L. plantarum* MPL16 was more efficient than the CRL1506 strain to improve *IFN-β* (24 h), *IFN-γ* (Figure 7A, Supplementary Figure S4A), and *Mx1* (Figure 7B). In addition, only the MPL16 strain increased *IFN-β* at h 6 post-infection compared to controls (Supplementary Figure S4A).

PRRSV infection also enhanced the expressions of *IL-1β*, *IL-8*, and *MCP-1* after 24 h (Figure 7E,F). In control infected pAMs, no changes in the expressions of *IL-6,* or *IL-27* were detected in comparison to non-infected cells both at 6 (Supplementary Figure S4C) and 24 (Figure 7E) h post-infection. *L. plantarum* MPL16 and CRL1506 treatments enhanced *IL-1β* at 6 h post-infection (Supplementary Figure S4C) but decreased this cytokine at h 24 (Figure 7E). Both strains augmented *IL-27* (6 h) and only *L. plantarum* MPL16 increased IL-6 and IL-8 at 6 h post-infection (Supplementary Figure S4C).

### 2.5. Combined Administration of Lactiplantibacillus plantarum Strains Is Not Better than Single Strains to Improve Antiviral Immunity in Porcine Alveolar Macrophages

**Figure 8:**
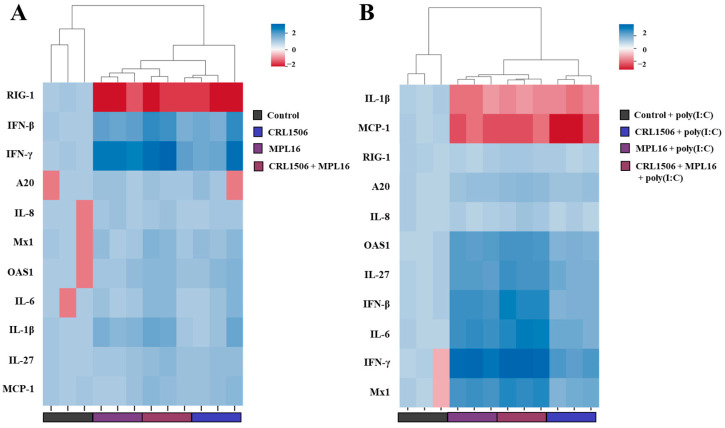
The effect of combined administration of *Lactiplantibacillus plantarum* CRL1506 and MPL16 on the cytokine profile of porcine alveolar macrophages induced by TLR3 activation. Porcine alveolar macrophages were stimulated with CRL1506, MPL16 or CRL1506 + MPL16 strains for 24 hours and then stimulated with poly(I:C) for 12 hours. The expressions of interferons, antiviral factors, the regulatory factor A20, and proinflammatory and regulatory cytokines genes were determined before (**A**) and after (**B**) poly(I:C) stimulation. Porcine alveolar macrophages without treatments (**A**) or not treated with lactobacilli and stimulated with poly(I:C) (**B**) were used as controls. Heatmap shows the variations in the expressions of immune factors of all experimental groups in relation to the control.

We evaluated whether the combination of *L. plantarum* MPL16 and CRL1506 was more effective than the single-strain administration to modulate antiviral immunity in pAMs. The CRL1506 + MPL16 treatment effectively enhanced the expressions of *IFN-β*, *IFN-γ* (Supplementary Figure S5A), *Mx1*, *OAS1* (Supplementary Figure S5B), *IL-1β*, and *IL-6* (Supplementary Figure S5C) in pAMs under non-inflammatory conditions. The combination of both lactobacilli also reduced *RIG-1* expression while no effects were observed for *A20* (Supplementary Figure S5A), *IL-27* (Supplementary Figure S5A), *IL-8* and *MCP-1* (Supplementary Figure S5D). The heat-map analysis of the effects of CRL1506, MPL16 and CRL1506 + MPL16 treatments on the transcriptomic response of pAMs in non-inflammatory conditions revealed no significant differences between the groups (Figure 8A).

We also observed that the combination of *L. plantarum* MPL16 and CRL1506 induced increases in *IFN-β*, *IFN-γ, A20* (Supplementary Figure S6A), *Mx1*, *OAS1* (Supplementary Figure S6B), *IL-6*, and *IL-27* (Supplementary Figure S6C) as well as decreases in *IL-1β* (Supplementary Figure S6B) and *MCP-1* (Supplementary Figure S6D) in pAMs stimulated with poly(I:C) compared to controls. The heat-map analysis comparing the effects of lactobacilli treatments on the transcriptomic response of pAMs after TLR3 activation showed a clear separation of the CRL1506 + MPL16 group from the control cells and the pAMs treated with CRL1506 alone (Figure 8B). In addition, a slight but significant difference (*p* < 0.05) was observed between MPL16 and CRL1506 + MPL16 groups.

**Figure 9:**
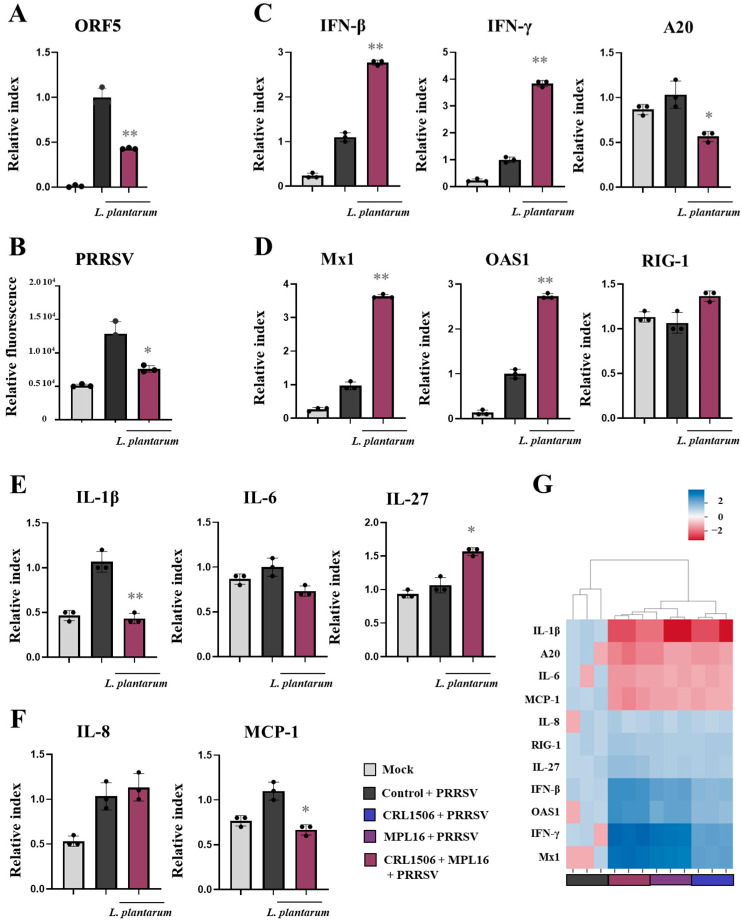
The effect of combined administration of *Lactiplantibacillus plantarum* CRL1506 and MPL16 on the immune response of porcine alveolar macrophages to Porcine Reproductive Respiratory Syndrome Virus (PRRSV) infection. Porcine alveolar macrophages were stimulated with CRL1506 + MPL16 strains for 24 h and then infected with PRRSV for 12 h. The expressions of viral gene (**A**) and antigens (**B**) as well as interferons (**C**), antiviral factors (**D**), the regulatory factor A20 (**C**), and proinflammatory and regulatory cytokines (**E**) and chemokines (**F**) genes were determined. Porcine alveolar macrophages not treated with lactobacilli and infected with PRRSV were used as controls. The expressions of immune factors in pAMs without any stimuli (mock) are shown. The results are shown as mean ± SD. Significant differences are shown compared to the PRRSV-infected control group at *p* < 0.05 (*) or *p* < 0.01 (**). Heatmap shows the variations in the expressions of immune factors of all experimental groups in relation to the control (**G**).

The combination of CRL1506 + MPL16 strains also improved the resistance of pAMs to PRRSV infection since the expression of *ORF5* (Figure 9A) and the viral antigens measured by IF (Figure 9B, Supplementary Figure S3) were significantly lower that infected-control cells. CRL1506 + MPL16 treatment improved *IFN-β*, *IFN-γ*, (Figure 9C), *Mx1*, *OAS1* (Figure 9D) and *IL-27* (Figure 9E) in PRRSV-infected pAMs, while no effect was observed for *RIG-1* and *IL-6*. In addition, the combination of CRL1506 + MPL16 strains reduced the expressions of *A20* (Figure 9C) and *IL-1β* (Figure 9E) in infected pAMs. As observed for poly(I:C) stimulation, the heat-map analysis comparing the lactobacilli treatments on the transcriptomic response of pAMs after PRRSV infection showed a clear separation of the CRL1506 + MPL16 group from the control cells and the pAMs treated with CRL1506 alone (Figure 9G). And although a trend toward a better response was observed for the combination of strains than for *L. plantarum* MPL16 alone, the difference was not statistically significant.

### 2.6. Lactiplantibacillus plantarum CRL1506 Is Better than MPL16 to Improve IFN-λ in Porcine Respiratory Epithelial Cells

**Figure 10:**
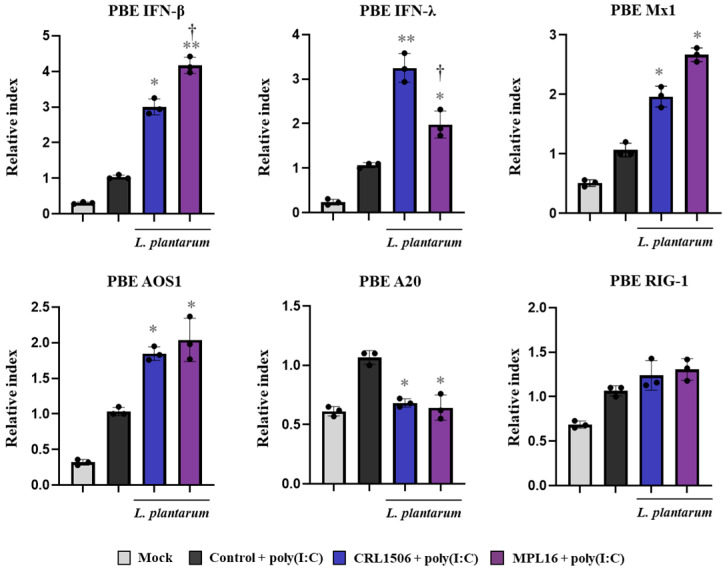
The effect of *Lactiplantibacillus plantarum* CRL1506 and MPL16 on the antiviral factors profile of porcine bronchial epithelial cells induced by TLR3 activation. Porcine bronchial epithelial cells (PBE cells) were stimulated with CRL1506 or MPL16 strains for 48 h and then stimulated with poly(I:C) for 6 h. The expressions of interferons, antiviral factors and the regulatory factor A20 genes were determined. PBE cells not treated with lactobacilli and stimulated with poly(I:C) were used as controls. The expressions of immune factors in PBE cells without any stimuli (mock) are shown. The results are shown as mean ± SD. Significant differences are shown compared to the poly(I:C)-stimulated control group at *p* < 0.05 (*) or *p* < 0.01 (**). Significant differences are shown compared to the CRL1506 strain administration at *p* < 0.05 (†).

We evaluated the effect of lactobacilli on the immune response of PBE cells triggered by TLR3 activation. The respiratory cells were stimulated with CRL1506 or MPL16 strains and then challenged with poly(I:C). The TLR3 agonist was able to increase the expression of all the antiviral factors evaluated in PBE cells compared to basal conditions (Figure 10). The expressions of *IFN-β*, *IFN-λ*, *Mx1*, and *OAS1* in lactobacilli-treated cells were significantly higher than controls. In addition, the regulatory factor *A20* was lower in CRL1506- and MPL16-treated cells than controls while no differences were found between the groups when *RIG-1* expression was analyzed. Of note, *L. plantarum* MPL16 was more efficient than the CRL1506 strain to augment *IFN-β* expression while the opposite was true for *IFN-λ* (Figure 10).

**Figure 11:**
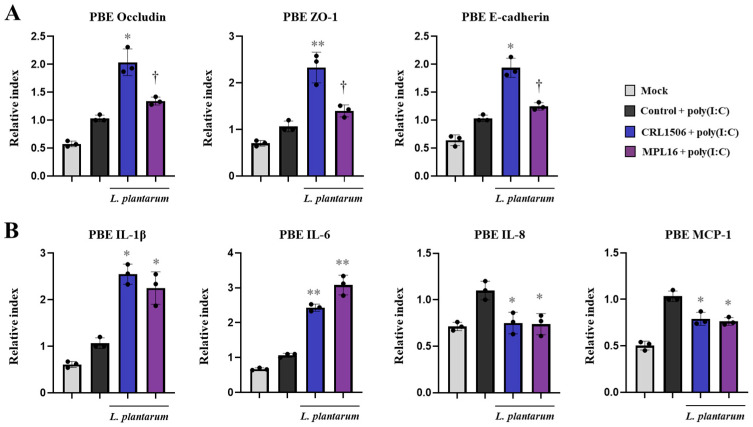
The effect of *Lactiplantibacillus plantarum* CRL1506 and MPL16 on the tight junctions and cytokine profile of porcine bronchial epithelial cells induced by TLR3 activation. Porcine bronchial epithelial cells (PBE cells) were stimulated with CRL1506 or MPL16 strains for 48 h and then stimulated with poly(I:C) for 6 h. The expressions of tight junctions (**A**) and cytokine (**B**) genes were determined. PBE cells not treated with lactobacilli and stimulated with poly(I:C) were used as controls. The expressions of immune factors in PBE cells without any stimuli (mock) are shown. The results are shown as mean ± SD. Significant differences are shown compared to the poly(I:C)-stimulated control group at *p* < 0.05 (*) or *p* < 0.01 (**). Significant differences are shown compared to the CRL1506 strain administration at *p* < 0.05 (†).

We also determined changes in the expression of tight junctions proteins in PBE cells after the TLR3 activation (Figure 11A). Poly(I:C) administration slightly but significantly increased the expressions of occludin, ZO-1 and E-cadherin in PBE cells. Interestingly, only *L. plantarum* CRL1506 improved the expression of the three proteins while the values in PBE cells treated with the MPL16 strain were not different from controls. Activation of TLR3 signaling also increased the expressions of *IL-1β*, *IL-6*, *IL-8* and *MCP-1* in PBE cells (Figure 11B). Both lactobacilli treatments were equally effective in reducing *IL-8* and *MCP-1* and increasing *IL-1β* and *IL-6*.

**Figure 12:**
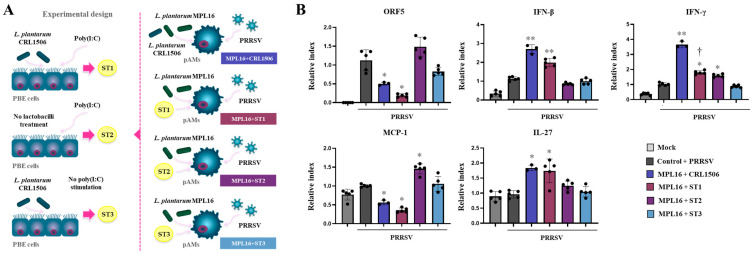
The effect of *Lactiplantibacillus plantarum* CRL1506 supernatant and MPL16 on the immune response of porcine alveolar macrophages to Porcine Reproductive Respiratory Syndrome Virus (PRRSV) infection. Culture supernatants were obtained from cultures of porcine bronchial epithelial cells (PBE cells) stimulated with the CRL1506 strain and poly(I:C) (ST1), poly(I:C) (ST2) or CRL1506 (ST3). Porcine alveolar macrophages (pAMs) were stimulated with MPL16 + CRL1506 strains, or MPL16 and the distinct supernatants for 24 h and then infected with PRRSV for 12 h (**A**). The expressions of interferons, MCP-1 and IL-27 genes were determined (**B**). pAMs not treated with lactobacilli or supernatants and infected with PRRSV were used as controls. The expressions of immune factors in pAMs without any stimuli (mock) are shown. The results are shown as mean ± SD. Significant differences are shown compared to the PRRSV-infected control group at *p* < 0.05 (*) or *p* < 0.01 (**). Significant differences are shown compared to the MPL16 + CRL1506 administration at *p* < 0.05 (†).

Given the differential changes induced by *L. plantarum* CRL1506 in PBE cells after TLR3 activation, we aimed to assess whether immune factors produced by CRL1506-treated PBE cells could influence the effect of *L. plantarum* MPL16 on pAMs responses to PRRSV infection. To this end, culture supernatants from CRL1506-treated, poly(I:C)-stimulated PBE cells (ST1) were collected and co-administered with MPL16 to stimulate pAMs, which were then infected with PRRSV (Figure 12A). Supernatants from PBE cells not exposed to lactobacilli but stimulated with poly(I:C) (ST2) and from cell treated only with *L. plantarum* CRL1506 (ST3) served as controls.

It was observed that the treatment of pAMs with the MPL16 strain and the ST1 was as effective as CRL1506 + MPL16 to reduce the replication of the virus, enhance the expression of *IFN-β* and *IL-27* and reduce the expression of *MCP-1* (Figure 12B). Of note, althougth MPL16 + ST1 improved *IFN-γ* expression in pAMs in response to PRRSV, the levels did not reach the observed for CRL1506 + MPL16 treatment. No effect was observed for the supernatant obtained from PBE cells stimulated only with *L. plantarum* CRL1506 (Figure 12B). In contrast, the ST2 obtained after the stimulation of PBE cells with poly(I:C) only increased the expression of *IFN-γ* and *MCP-1*, but not affected *IL-27*, indicating a proiflammatory action.

## 3. Discussion

Research from the last decade has shown that nasally delivered immunomodulatory microorganisms can impact the host’s resistance against viral infections [7,8,11]. Importantly, since innate defenses are modulated by lactobacilli, their effect is independent of the type of virus infecting the respiratory tract and therefore they are able to increase the resistance to different type of viruses. In this regard, studies demonstrated that the nasal priming with immunomodulatory lactobacilli can improve the response of mice to IFV [11], RSV [7,8] and PVM [9,10] infections. In most of these studies single immunobiotic strain administrations were used. To the best of our knowledge, this work is the first study to comparatively evaluate the nasal priming with two immunobiotic strains—administered individually or in combination—on TLR3- and RSV-mediated respiratory immune responses in mice.

In our hands, the nasal priming with individual lactobacilli or the combination of both significantly reduced RSV replication and lung tissue injury after the viral challenge or the activation of TLR3-signaling in the respiratory tract. The protective effect was related to a differential modulation of IFNs, antiviral factors as well as inflammatory and regulatory cytokines. In line with our previous results [7,8,11], lactobacilli treatment increased the production of IFN-β and IFN-γ in the respiratory tract with the subsequent improvement of antiviral factors expressions. In addition, when the population of mAMs was evaluated, a remarkable improvement of the antiviral factors *IFNs*, *OAS1*, *Mx2*, *RNAseL*, and *IFITM3* was observed in mice treated with lactobacilli compared to the control group. Timely induction of type I IFNs, interferon-stimulated genes, and IFN-γ is crucial for protection against RSV. AMs—among the first immune cells to face viruses at the respiratory mucosa—contribute to antiviral defences by producing type I IFNs and IFN-γ [19], which facilitates pathogen elimination [20]. OAS1 restricts protein synthesis and viral growth by degrading viral RNA, while IFITM3 inhibits early steps of the viral cycle; both factors impede RSV replication [21,22]. Likewise, RNAseL degrades viral RNA [23], thereby enhancing protection against RSV [24]. Increased Mx2 levels in the respiratory tract have also been linked to resistance to RSV [25]. Accordingly, up-regulation of these antiviral mediators aligns with the improved RSV clearance observed in lactobacilli-treated animals. Furthermore, the quantitative differences in IFNs production and antiviral factor expression between animals treated with a single strain and those given the two-strain combination could explain the CRL1506 + MPL16 mixture’s superior protection against RSV and its enhanced modulation of TLR3-mediated innate immunity.

During respiratory viral infections, AMs must not only mount appropriate antiviral defenses but also tightly regulate inflammation to promote virus clearance while preventing inflammation-induced tissue injury. We previously showed that nasal stimulation of mice with immunobiotic lactobacilli enhanced the capacity of mAMs to produce IL-27 and IL-6 upon TLR3 activation. The IL-6 and IL-27 produced by mAMs promoted IL-10-secreting regulatory T cells, which helped to control lung inflammation [7,8]. Similarly, mice nasally treated with *L. plantarum* CRL1506, MPL16 or the combination of CRL1506 + MPL16 had improved levels of IL-6, IL-27 and IL-10 in the respiratory tract and in mAMs after poly(I:C) challenge or RSV infection. Moreover, the levels of inflammatory cytokines and chemokines in lactobacilli-treated mice were lower than controls, indicating the ability of immunobiotic treatments to modulate the balance of pro- and anti-inflammatory mediators and thus protect against lung damage, as evidenced by the lower levels of all injury markers evaluated. Of note, the CRL1506 + MPL16 combination was more effective than either strain alone at reducing lung tissue damage through the modulation of cytokine and chemokine production.

This work shows, for the first time, that the nasal priming with a combination of immunobiotic strains is more effective than single-strain administration at modulating respiratory antiviral immunity in vivo. We then sought to elucidate the mechanisms underlying the synergistic effect between the two *L. plantarum* strains. Potential benefits of multi-strain formulations in the context of infections include synergistic interactions among constituent strains—such as enhanced mucosal adhesion or greater pathogen suppression. As it was mentioned before, the administration of *L. rhamnosus* GG and *B. lactis* Bb12 together was more efficient for *H. pylori* eradication than the GG strain alone [13]. It was proposed that this synergistic effect was related to enhanced mucus binding adhesion of *B. lactis* Bb12 when combined with the GG strain [26]. An additional option, particularly for immunobiotic strains, is the complementary actions on the immune system. It has been shown that the interaction of different strains of commensal microorganisms can affect the cytokine profile produced by the immune system, both qualitatively and quantitatively. The single strain *Lactobacillus helveticus* R52 prevented antibiotic-associated diarrhea; however, adding other strains such as *L. rhamnosus* R11 and *B. longum* I-175 eliminated the protection of the intestinal mucosa. This loss of effect was explained by the reduced cytokine production observed for the mixture compared with the cytokine levels induced by the individual R52 strain [27]. Considering this background and our results in mice that showed the superior ability of mAMs treated with the combination of CRL1506 + MPL16 compared to individual strains to express IFNs, antiviral factors and inflammatory and regulatory cytokines, our first hypothesis was that both strains would exert a synergistic effect on these immune cells. To study this hypothesis, we performed in vitro experiments with individual and combined lactobacilli strains in a pAMs cell line stimulated with the TLR3 agonist poly(I:C) or challenged with PRRSV, a virus that efficiently replicates in pAMs [28].

We evaluated whether *L. plantarum* CRL1506, MPL16 or the combination of both increased the resistance of pAMs to PRRSV infection by the differential modulation of their immune response. In our study, the infection of pAMs with PRRSV significantly increased the expression of *IFN-β* and *IFN-γ*, which is in agreement with previous studies showing augmented IFN-α and IFN-γ levels in the cell line 3D4/21 (alveolar macrophages of porcine origin), after the challenge with PRRSV [28]. Of note, it was reported that Meishan and Tongcheng (TC) pigs are able to produce higher IFN-γ levels than Large White (LW) pigs, which are more susceptible to PRRSV infection [29,30,31], indicating the important role of IFN-γ-mediated cellular immune response in the resistance to PRRSV infection. On the other hand, studies have demonstrated that PRRSV is sensitive to the action of IFN-α and IFN-β [32]. In vitro and in vivo studies showed that the efficient infection of pAMs with PRRSV induced marginal amounts of type I IFNs production [28,33] and that this effect resulted also in a weak IFN-γ response [28]. Both, IFN-β and IFN-γ stimulate the production of antiviral factors to counteract viral replication including Mx1 and OAS1. It was reported that both OAS1 [34] and Mx1 [35] have inhibitory effects against PRRSV helping to restrict viral replication and diminish cytopathogenic effects. In this work, we demonstrated that the pretreatment of pAMs with *L. plantarum* CRL1506 or MPL16 significantly increased the expression of *IFN-β*, *IFN-γ*, and the antiviral factors *Mx1* and *OAS1* and reduced *A20* in response to PRRSV infection, an effect that correlated with lower viral replication. Surprisingly, the treatment of pAMs with CRL1506 + MPL16 was as effective as the single-strain administration to modulate immunity and improve resistance to PRRSV.

In pigs infected with PRRSV, heightened expression of *TNF-α*, *IL-1β*, *IL-6*, and *IL-8* has been documented in lung tissue [36,37] and also detected systemically in peripheral blood [38]. These inflammatory cytokines foster an antiviral milieu in bystander cells that curtails PRRSV replication, promote cytolysis of infected targets, and support cell survival [39,40]. Lung-resident immune cells, notably macrophages, further amplify inflammation by producing mediators that draw monocytes and neutrophils into sites of infection, thereby aiding viral clearance [41]. Yet, without proper control, this same response can inflict collateral damage on pulmonary tissue [42]. Consistent with this, highly pathogenic PRRSV variants provoke disproportionately greater IL-6 [37] and IL-1β [43] production in vivo and in vitro than low-virulence strains. Moreover, virulent PRRSV replicates more efficiently in the lungs, elicits stronger inflammatory cytokine responses, and produces more severe pneumonia than less pathogenic counterparts [37]. In line with these previous results, we showed that pAMs stimulated with poly(I:C) or challenged with PRRSV increased *IL-1β*, *IL-6*, *IL-8* and *MCP-1*, and that lactobacilli pretreatments differentially modulated the expression of this cytokines. Again, the combination of CRL1506 + MPL16 was as effective as the single-strain to modulate inflammation in pAMs.

These results clearly indicated that our initial hypothesis was incorrect: administering the two lactobacilli strains did not enhance macrophage responses beyond those induced by the individual strains. We therefore advanced a second hypothesis considering that, in vivo, immunobiotic activity reflects their effects on the complex interaction network linking immune and non-immune cells in the respiratory tract. Among the first cells to encounter inhaled viruses are epithelial cells, which can restrict replication by producing antiviral mediators and by recruiting and activating immune cells [44]. Notably, immunobiotics have been shown to modulate the antiviral responses of respiratory epithelial cells [12,45]. It was demonstrated that *L. rhamnosus* D3189 reduce RSV shedding in primary nasal epithelial cells through the modulation of antiviral and inflammatory responses. Nasal epithelial cells from healthy adult donors in vitro stimulated with the D3189 strain had improved production of IFN-β in response to RSV infection [45]. Furthermore, in the human respiratory Calu-3 cells we previously showed that *L. plantarum* MPL16 and CRL1506 modulated the production of IFN-β, and antiviral factors in response to poly(I:C) and SARS-CoV-2 challenges [12]. Therefore, we hypothesized that the two lactobacilli would exert a distinct effect on the respiratory epithelial cells and that the qualitative and/or quantitative differences in the cytokines produced by these cells would act on the AMs, exerting a differential effect.

**Figure 13:**
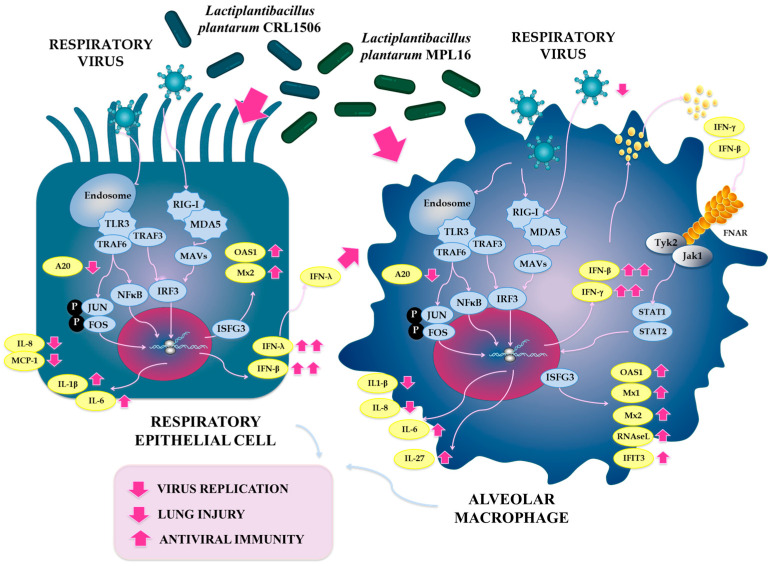
Proposed mechanism for the effect of the combined administration of *Lactiplantibacillus plantarum* CRL1506 and MPL16 on the respiratory antiviral immune response and the resistance against viruses.

Our results showed that PBE cells stimulated with *L. plantarum* CRL1506 or MPL16 had a distinct expression of tight junctions proteins, IFNs, antiviral factors and inflammatory cytokines in response to poly(I:C) challenge compared to non-lactobacilli treated cells. More importantly, the transcriptomic response of CRL1506-treated PBE cells was different to the observed for the MPL16 strain, particularly in the expression of tight junctions, *IFN-β* and *IFN-λ*. The different ability of lactobacilli strains to impact respiratory epithelial cells could explain the synergistic effect observed in vivo. Respiratory epithelial cells do not produce IFN-β and IFN-λ in a fixed ratio. The balance depends on the cell type, the PRR engaged, stimulus dose, and viral factors. There is evidence that the same stimulus can drive distinct amounts/kinetics of IFN-β versus IFN-λ in the respiratory epithelium [46,47,48]. Studies performed in mice with IFV and poly(I:C) showed that IFN-λ is often the predominant interferon made by respiratory epithelial cells, exceeding IFN-β under the same conditions [46]. Similarly, in response to RSV infection, IFN-λ and not type I IFNs, are the primary IFN produced by nasal epithelium [47]. IFN-λ in epithelia are tuned by pathways like the TLR3–IRF1 axis, and type I and III IFNs show different induction dynamics and downstream transcriptional programs even when triggered by the same stimuli like poly(I:C) [48,49]. On the other hand, it was showed that human airway epithelial cells infected with IFV produce IFN-λ, and that supernatants from these infected cells induced the expression of antiviral factors in human AMs [50]. The effect of the supernatants of respiratory epithelial cells disappeared when IFN-λ signaling (IFNLR1) was knocked out in AMs. The work also found that pretreating human AMs with IFN-λ directly reduced IFV infection, indicating a functional antiviral boost. Then, the superior in vivo protection against RSV in mice achieved by the combinantion of *L. plantarum* CRL1506 and MPL16 can be explained by the complementary actions of the two strains: CRL1506 and MPL16 robustly modulate AMs, while CRL1506 acts more efficiently on epithelial cells, resulting in an improved increase in IFN-λ that further augments macrophage antiviral activity (Figure 13). The molecular mechanisms involved in the greater capacity of *L. plantarum* CRL1506 to increase IFN-λ in the respiratory epithelium compared to the MPL16 strain is an interesting topic for future research.

Of note, no studies have investigated the potential application of lactobacilli strains to improve the resistance against PRRSV in pigs or in porcine in vitro systems. Here, we showed that *L. plantarum* CRL1506 and MPL16, single or combined, enhanced the resistance of pAMs to PRRSV infection. The nasal administration of immunobiotics in animals is currently underexplored, especially for the prevention of viral infections. The comparative studies of the nasopharyngeal microbiota of healthy cattle and cattle with bovine respiratory disease showed a greater relative abundance of *Lactobacillaceae* in healthy individuals [51]. Similarly, lactobacilli were more abundant among the nasopharyngeal and tracheal microbial communities in helathy feedlot cattle compared with those suffering from bronchopneumonia [52]. Interestingly, the nasal administration of six lactobacilli strains selected by their capacities to inhibit and exclude respiratory pathogens and to modulate immune responses in bovine turbinate cells improved respiratory health of cattle [53,54]. On the other hand, it was reported that antibiotic-induced dysbiosis of the nasal microbiome in dogs increase epithelial barrier disruption and alter immune response to IFV infection [55]. The work also demonstrated that *L. plantarum* C123, originally isolated from canine nasal cavity, has a remarkable capacity to modulate the antiviral immune response and the resistance to IFV infection in vitro. No studies have documented the potential benefits of nasally administered immunobiotic lactobacilli on the respiratory health of pigs. Then, the results of this work suggest that the strains CRL1506 and/or MPL16, nasally administered, could be an excellent alternative to prevent or reduce the severity of PRRSV infections in pigs.

## 4. Materials and Methods

### 4.1. Microorganisms

*Lactiplantibacillus plantarum* CRL1506 belong to the CERELA-CONICET (San Miguel de Tucuman, Argentina) culture collection. *Lactiplantibacillus plantarum* MPL16 belong to the culture collection of the Laboratory of Animal Food Function of the Graduate School of Agricultural Science of Tohoku University (Sendai, Japan). Lactobacilli were seeded by streaking on Man-Rogosa-Sharpe (MRS) agar (Sigma, Detroit, MI, USA) for 24 h. After growth, a colony was incubated on 1 mL of MRS broth for 16 h for further steps.

The PRRSV EDRD-1 strain was provided by the Division of Infectious Animal Disease Research, National Institute of Animal Health, National Agriculture and Food Research Organization (Tsukuba, Japan). The virus was propagated in porcine alveolar macrophages (PAM-KNU cell line, described below), aliquoted and stored at −80 °C until use. The PRRSV titration was performed as described previously [56].

### 4.2. Animals and Feeding Procedures

Male BALB/c mice (5 weeks old) were obtained from the closed colony at CERELA-CONICET (San Miguel de Tucumán, Argentina) and kept in plastic cages at ambient temperature. For each parameter, groups of seven animals were used. Lactobacilli were washed, resuspended in PBS, and administered intranasally to distinct mouse groups. Single strains and a 1:1 mixture were delivered by the nasal route at $10^{8}$ cells/mouse/day for five consecutive days. The optimal treatment of each strain to induce immunomodulatory activities were previosuly evaluated using different doses and periods of administration [7,11]. Control group received only PBS. Throughout the study, all mice were fed a balanced diet ad libitum. The research was conducted in strict compliance with the Guide for Care and Use of Laboratory Animals and was approved by CERELA’s Ethical Committee under the protocols CRL-CICUAL-IBT-2024/3A and CRL-CICUAL-IBT-2024/7A.

### 4.3. Intranasal Administration of Poly(I:C) and RSV Infection

Forty-eight hours after lactobacilli treatment, mice were challenged with the TLR3 agonist poly(I:C) (Sigma-Aldrich, St. Louis, MO, USA). To induce TLR3-mediated lung inflammation, 100 µL PBS containing 250 µg poly(I:C) (10 mg/kg body weight) was applied dropwise to the nares [7,11]. Control animals received 100 µL PBS alone. Poly(I:C) was administered as three doses, each separated by a day interval.

Human RSV strain A2 was propagated in Vero cells following standard methods [7]. Monolayers in DMEM were infected at MOI = 1 using a 5 mL inoculum for 3 h at 37 °C in 5% CO_2_. After adsorption, 7 mL DMEM with 10% FBS, 0.1% penicillin–streptomycin, and 0.001% ciprofloxacin were added, and cultures were incubated until extensive syncytium formation was evident. Cells were harvested by scraping, disrupted by sonication, and clarified by centrifugation (700× *g*, 10 min, 4 °C). The viral supernatant was purified on a sucrose density gradient and stored at −80 °C in 30% sucrose.

For the in vivo challenge, mice received an intranasal dose of RSV at 10^6^ PFU exactly 24 h after the final nasal priming with lactobacilli. Forty-eight hours post-infection, we quantified viral burden in the lungs and evaluated pulmonary injury. RSV loads were measured by an immunoplaque assay following established protocols [7]. Briefly, lungs were homogenized, clarified, and the supernatants were distributed in triplicate onto Vero cell monolayers. After a 3 h adsorption period, the inoculum was replaced with fresh DMEM supplemented with 10% FBS, 0.1% penicillin–streptomycin, and 0.001% ciprofloxacin. When extensive syncytium formation became evident, cells were fixed with chilled acetone:methanol (60:40), probed with RSV-specific anti-F and anti-G antibodies, and then with horseradish peroxidase-conjugated secondaries. Plaques were developed using a DAB substrate kit (Sigma-Aldrich, St. Louis, MO, USA) and titers were reported as log10 PFU/g of lung tissue.

### 4.4. Lung Injury Parameters

BAL samples were collected following established methods [7,8]. In cell-free BAL, lung damage was assessed by measuring protein and albumin (markers of broncho-alveolar–capillary leak) and LDH activity (cytotoxicity index). Albumin was quantified by a bromocresol green–based colorimetric assay (Wiener Lab, Buenos Aires, Argentina); total protein by BCA (Pierce Biotechnology Inc., Rockford, IL, USA); and LDH activity (U/L) by NADH generation using Wiener Lab’s standard protocol. In addition, soluble E-cadherin was measured the serum and BAL samples with the Mouse E-Cadherin (CDH1) ELISA Kit (Invitrogen, ThermoFisher Scientific, Waltham, MA, USA).

### 4.5. Cytokine Concentrations in BAL

Commercially available ELISA kits were used to assess the levels of TNF-α, IFN-β, IFN-γ, IL-1β, IL-6, IL-10, IL-27, MCP-1, and KC (R&D Systems, Minneapolis, MN, USA) in BAL samples. For these procedures, the manufacturer’s instructions were followed.

### 4.6. Alveolar Macrophage Primary Cultures

Following published protocols [8], primary cultures of murine alveolar macrophages (mAMs) were generated from BAL fluid. Mice were lavaged with 1 mL pre-warmed sterile PBS containing 5 mM EDTA; collected cells were washed twice in PBS and suspended in RPMI 1640 plus 10% FBS, 1 mM L-glutamine, and 100 U/mL penicillin–streptomycin. Cells were plated in 24-well plates at 1 × 10^5^ cells per well, incubated 2 h at 37 °C and 5% CO_2_ for adherence, and then cultured an additional 24 h under the same conditions. Macrophages were stimulated with poly(I:C) at 50 µg/mL, and after 12 h, mRNA was extracted to quantify cytokine and antiviral factor transcripts by RT-qPCR.

### 4.7. Porcine Bronchial Epithelial and Macrophages Cell Lines

The porcine alveolar macrophages cell line PAM-KNU-hTERT (Cat. No. T0741-C, ABM, Richmond, BC, Canada) derived from the lung of a euthanized 8-week-old specific pathogen free (SPF) pig was used. The PAM-KNU cells were cultured in tissue culture flask (Corning, NY, USA) with Roswell Park Memorial Institute (RPMI)-1640 medium (FUJIFILM Wako Pure Chemical Corporation, Tokyo, Japan) containing 10% of fetal calf serum (FCS) and 1% of penicillin-streptomycin solution. Porcine alveolar macrophages (pAMs) were incubated at 37 °C with 5% CO_2_.

The PBE cell line used in this study was derived from the bronchial tissue of a 7-day old piglet as described before [44]. PBE cells were cultured in DMEM (GIBCO, Grand Island, NY, USA), with 10% FCS and 1% penicillin/streptomycin at 37 °C with 5% CO_2_ in a humidified atmosphere (Thermo Fisher Scientific, Waltham, MA, USA; CO_2_ incubator).

### 4.8. Immunomodulatory Capacity of Lactobacilli in Porcine Cells

The pAMs were seeded at a concentration of 1 × 10^5^ cells/well on a 24-well tissue culture plate during 24 h. Subsequently, cells were stimulated with the lactobacilli utilizing a MOI of 10, and incubated at 37 °C with 5% CO_2_ during 24 h. Then, pAMs were stimulated with poly(I:C) (1 µg/mL) or infected with PRRSV at a MOI of 0.05.

The PBE cells were seeded at a concentration of 1 × 10^4^ cells/well on a 24-well tissue culture plate. The cells were stimulated with the lactobacilli utilizing a MOI of 10, and incubated at 37 °C with 5% CO_2_. Then, PBE cells were stimulated with poly(I:C) (1 µg/mL).

### 4.9. Immunofluorescence (IF)

The pAMs were seeded at a concentration of 1 × 10^4^ cells/well on a 96 well plate. After 24 h of incubation, the cells were stimulated with lactobacilli and infected with PRRSV as described before. Subsequently, pAMs were washed once with PBS, and fixed with acetone 80% for 5 min at 4 °C. The acetone was discarded, and the plate was let dry for 1 h. The cells were incubated with anti-PRRSV antibody SR30A (RTI, LLC) diluted 1:1000 in blocking buffer (1% goat serum on PBS). After 1 h of incubation, the wells were washed five times with PBS, and the alexa fluor 488-conjugated affinipure anti-mouse IgG (Sigma-Aldrich, MA, USA) was added and incubated for 1 h. The plate was washed five times with PBS and 1–2 drops of DAPI were added. The BZ-X800 all-in-one microscope (KEYENCE, Osaka, Japan) was used for the observation of samples.

### 4.10. RT-qPCR

Total RNA was isolated from mouse lungs, mAMs, and pAMs collected at the specified time points using TRIzol Reagent (Invitrogen, Carlsbad, CA, USA). cDNA was synthesized with the PrimeScript^TM^ RT reagent Kit with gDNA Eraser (Perfect Real Time; Takara Bio, Kusatsu, Shiga, Japan) according to the manufacturer’s protocol. RT-qPCR was carried out on a CFX Connect Real-Time System (Bio-Rad, Hercules, CA, USA) using TB Green Premix Ex Taq II (Takara Bio). Primers used to assess immune factor expression in mice have been described previously [8]. Cycling conditions were: 50 °C for 2 min; 95 °C for 2 min; then 40 cycles of 95 °C for 15 s, 60 °C for 30 s, and 72 °C for 30 s.

Expression levels were normalized using β-actin as a reference to account for variations in total cDNA among samples. The primers utilized to evaluate changes in the expression of immune factors in porcine cells were described previously [44]. For the expression of ORF5 PRRSV the following primers were used: *ORF5* forward TCTGGACACTAAGGGCAGACTC, reverse GGAACCATCAAGCACAACTCTC. The amplification conditions were 95 °C for 30 s, 40 cycles at 95 °C for 5 s, and then 60 °C for 30 s. β-actin was used as an internal control to normalize genes expressions.

### 4.11. Statistical Analysis

Statistical analysis was performed with GraphPad Prism v8 (GraphPad Software, San Diego, CA, USA). The results are expressed as mean ± standard deviation (SD). Verification of the normal distribution of data was performed, and 2-way ANOVA was used. Tukey’s test, for pairwise comparisons of the means, was utilized to evaluate for differences between the groups. Differences were considered significant at *p* < 0.05.

## 5. Conclusions

A fundamental question in probiotic research is whether single-strains outperform—or are comparable to—multi-strain formulations in terms of efficacy. While several single-strain of immunobiotics have evidence-based benefits, the complexity of the intestinal microbiome has led to the hypothesis that combining multiple strains may be more effective for restoring microbial balance and modulating the immune system [13]. This hypothesis can be applied also to the nasally administered immunobiotics considering the recent advances that demonstrated an analogous role of the respiratory microbiota in the modulation of the immune system in the airways and the lungs [57]. Our results indicate that combining *L. plantarum* CRL1506 and MPL16 is more effective than using single strains to reduce RSV replication, protect the lung from TLR3-mediated inflammatory injury and modulate innate antiviral responses, particularly in AMs, in mice. In addition, our in vitro experiments demonstrated that lactobacilli combination also increased the resistance of porcine AMs to PRRSV infection through the modulation of innate immunity. The combined administration of both strains would be more efficient in modulating the complex interactions that exist between immune cells (e.g., AM) and non-immune cells (e.g., epithelial cells) in the respiratory tract, in favor of the host’s health. The results from the animal models of this work furnish the scientific basis for proposing future human trials to assess the efficacy of the CRL1506 + MPL16 combination in improving respiratory antiviral immunity.

## Data Availability

The data presented in this study are available throughout the article.

## Supplementary Materials

The following supporting information can be downloaded at: https://www.mdpi.com/article/10.3390/ijms262010079/s1.

## Abbreviations

2′-5′-oligoadenylate synthetase 1 (OAS1), Alveolar macrophages (AMs), Broncho-alveolar lavage (BAL), DExD/H-box helicase 58 (DDX58), Influenza virus (IFV), Interferon (IFN), Interleukin (IL), Keratinocyte-derived chemokine (KC), Lactate dehydrogenase (LDH), Man-Rogosa-Sharpe (MRS), Monocyte chemoattractant protein-1 (MCP-1), Murine alveolar macrophages (mAMs), Myxovirus resistance protein 1 (Mx1), Open reading frame 5 (ORF5), Pneumonia virus of mice (PVM), Polyinosinic:polycytidylic acid [poly(I:C)], Porcine alveolar macrophages (pAMs), Porcine reproductive and respiratory syndrome virus (PRRSV), Porcine respiratory epithelial cells (PBE cells), Respiratory syncytial virus (RSV), Severe acute respiratory syndrome coronavirus 2 (SARS-CoV-2), Toll-like receptor (TLR)-3, Tumor necrosis factor (TNF).
