# Supplementary material for: Combined Lactiplantibacillus plantarum CRL1506 and MPL16 Nasal Priming More Effectively Modulates Respiratory Antiviral Innate Immunity than Single Strains
Source: Int J Mol Sci. 2025 Oct 16;26(20):10079. doi: 10.3390/ijms262010079 (PMC12563780; doi:10.3390/ijms262010079)
Supplement: Supplementary file 1 [file ijms-26-10079-s001.zip › ijms-3922644-supplementary.pdf]

Supplementary Figures

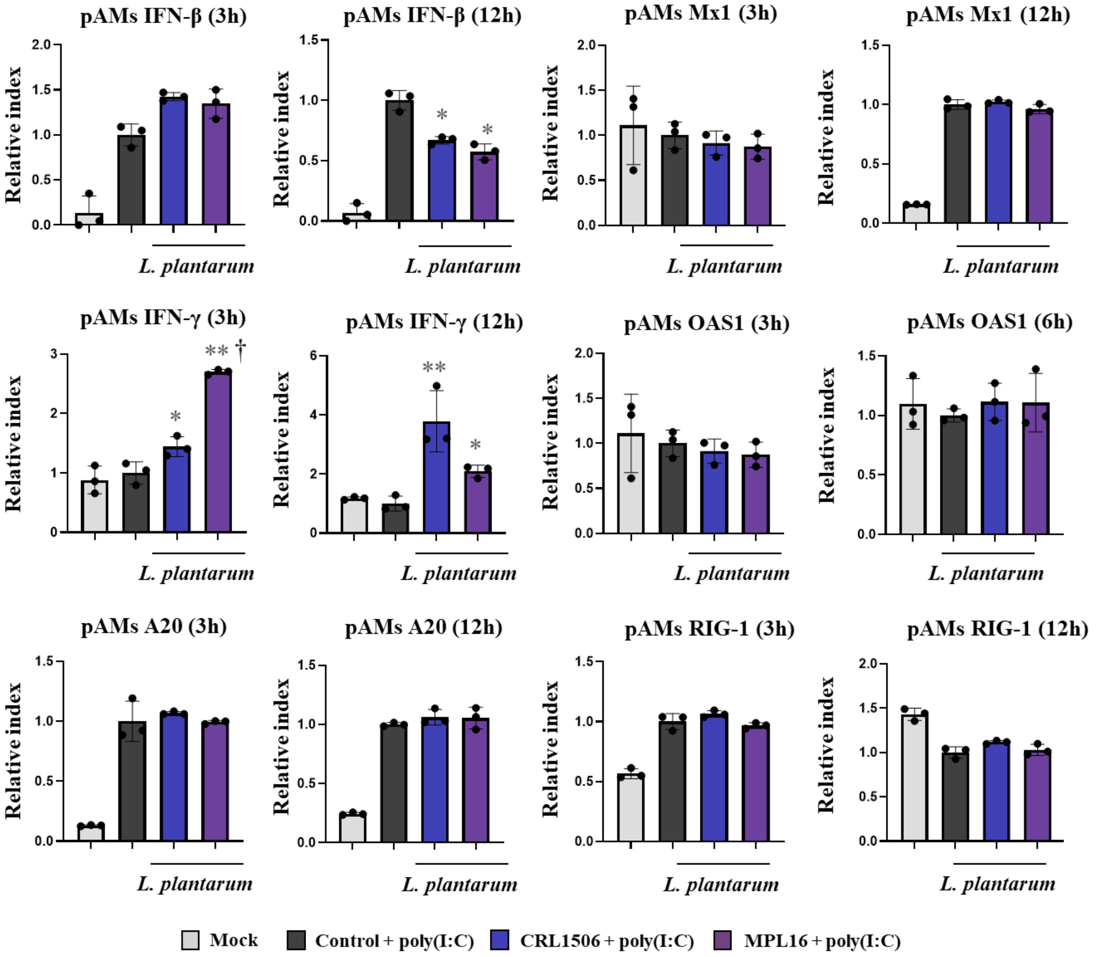

Figure S1. The effect of *Lactiplantibacillus plantarum* CRL1506 and MPL16 on the cytokine profile of porcine alveolar macrophages induced by TLR3 activation. Porcine alveolar macrophages (pAMs) were stimulated with CRL1506 or MPL16 strains for 24 hours and then stimulated with poly(I:C) for 3, 6 or 12 hours. The expressions of interferons, antiviral factors, and the regulatory factor A20 genes were determined. pAMs not treated with lactobacilli and stimulated with poly(I:C) were used as controls. The expressions of immune factors in pAMs without any stimuli (mock) are shown. The results are shown as mean ± SD. Significant differences are shown compared to the poly(I:C)-stimulated control group at $p < 0.05$ (*) or $p < 0.01$ (**). Significant differences are shown compared to the CRL1506 strain administration at $p < 0.05$ (†).

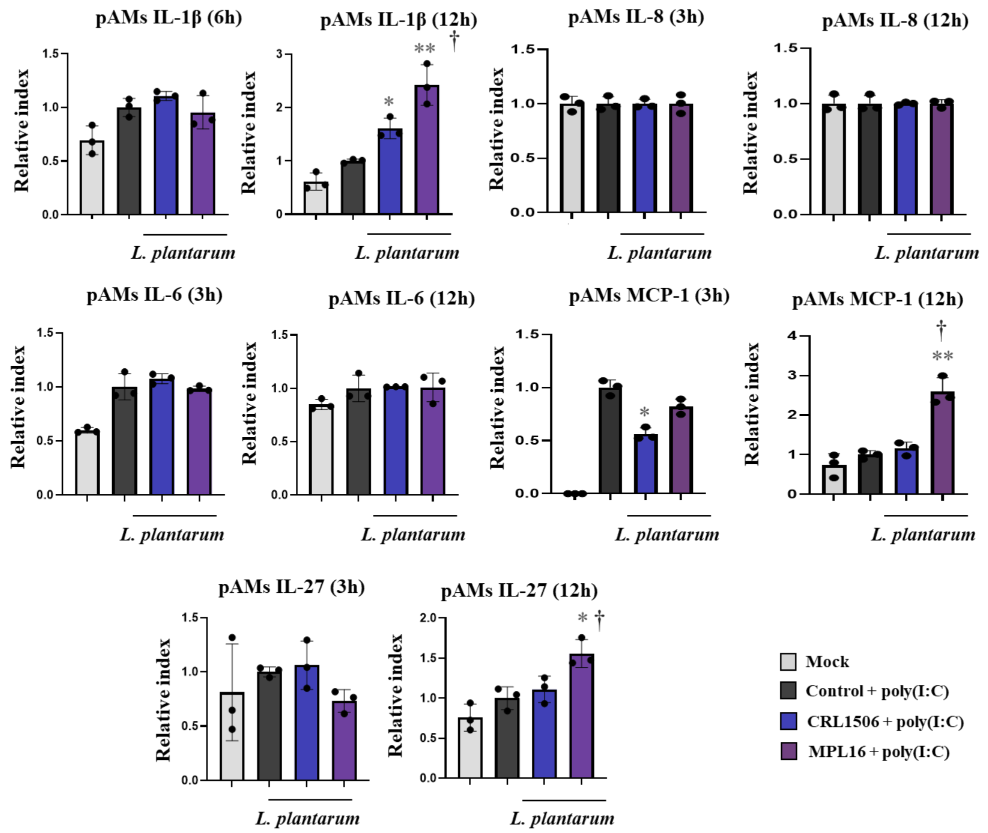

Figure S2. The effect of *Lactiplantibacillus plantarum* CRL1506 and MPL16 on the cytokine profile of porcine alveolar macrophages induced by TLR3 activation. Porcine alveolar macrophages (pAMs) were stimulated with CRL1506 or MPL16 strains for 24 hours and then stimulated with poly(I:C) for 3, 6 or 12 hours. The expressions of proinflammatory and regulatory cytokines genes were determined. pAMs not treated with lactobacilli and stimulated with poly(I:C) were used as controls. The expressions of immune factors in pAMs without any stimuli (mock) are shown. The results are shown as mean ± SD. Significant differences are shown compared to the poly(I:C)-stimulated control group at $p < 0.05$ (*) or $p < 0.01$ (**). Significant differences are shown compared to the CRL1506 strain administration at $p < 0.05$ (†).

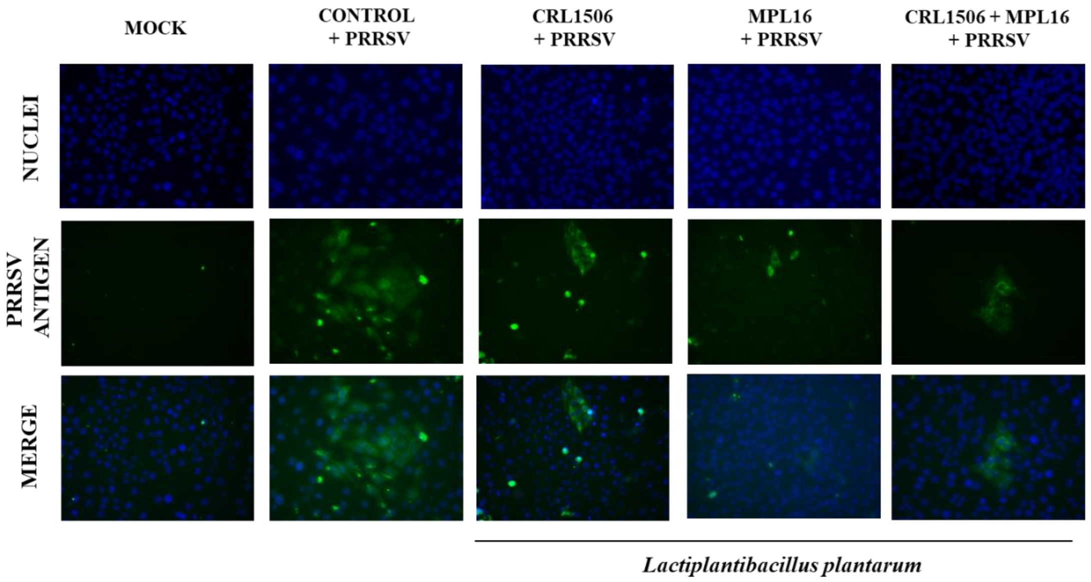

Figure S3. The effect of *Lactiplantibacillus plantarum* CRL1506 and MPL16 on the replication of Porcine Reproductive Respiratory Syndrome Virus (PRRSV) in porcine alveolar macrophages. Porcine alveolar macrophages (pAMs) were stimulated with CRL1506, MPL16 or CRL1506+MPL16 strains for 24 hours and then infected with PRRSV for 24 hours. The viral replication was determined by detecting virus antigens througth immunofluorescence. pAMs not treated with lactobacilli and infected with PRRSV were used as controls. The photos of pAMs without any stimuli (mock) are shown. The results are shown as mean ± SD. Significant differences are shown compared to the PRRSV-infected control group at p < 0.05 (*) or p < 0.01 (**).

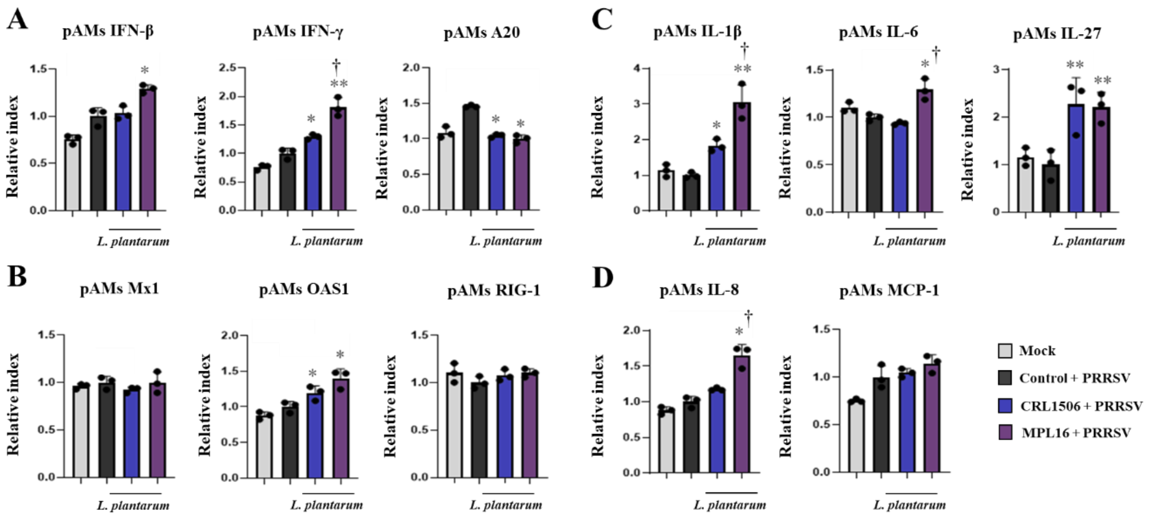

Figure S4. The effect of *Lactiplantibacillus plantarum* CRL1506 and MPL16 on the immune response of porcine alveolar macrophages to Porcine Reproductive Respiratory Syndrome Virus (PRRSV) infection. Porcine alveolar macrophages (pAMs) were stimulated with CRL1506 or MPL16 strains for 24 hours and then infected with PRRSV for 6 hours. The expressions of interferons, the regulatory factor A20 (**A**), antiviral factors (**B**), cytokines (**C**) and chemokines (**D**) genes were determined. pAMs not treated with lactobacilli and infected with PRRSV were used as controls. The expressions of immune factors in pAMs without any stimuli (mock) are shown. The results are shown as mean ± SD. Significant differences are shown compared to the PRRSV-infected control group at p < 0.05 (*) or p < 0.01 (**). Significant differences are shown compared to the CRL1506 strain administration at p < 0.05 (†).

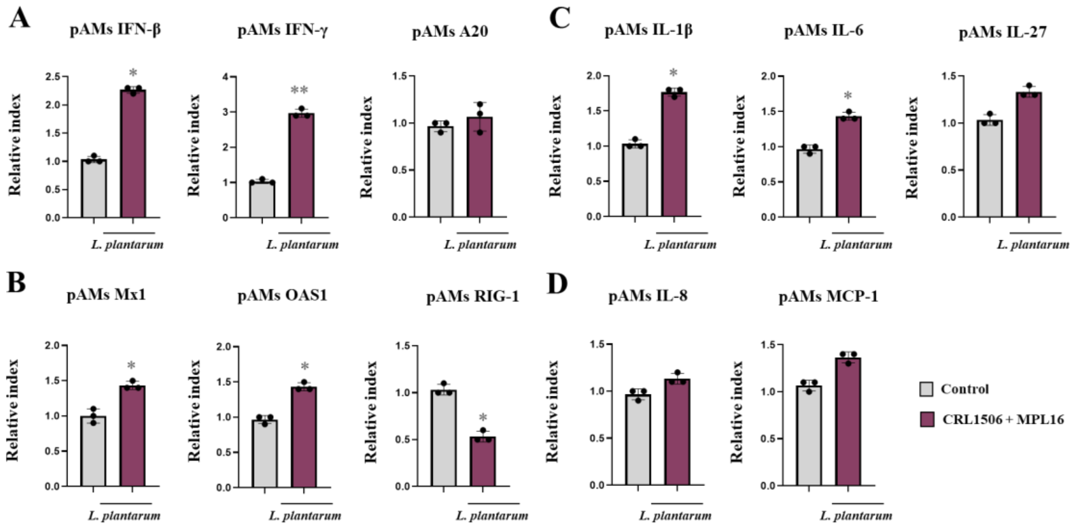

Figure S5. The effect of combined administration of *Lactiplantibacillus plantarum* CRL1506 and MPL16 on the cytokine profile of porcine alveolar macrophages. Porcine alveolar macrophages (pAMs) were

stimulated with CRL1506+MPL16 strains for 24 hours and then the expressions of interferons, antiviral factors, the regulatory factor A20, and proinflammatory and regulatory cytokines genes were determined. pAMs not treated with lactobacilli were used as controls. The results are shown as mean ± SD. Significant differences are shown compared to the poly(I:C)-infected control group at $p < 0.05$ (*) or $p < 0.01$ (**).

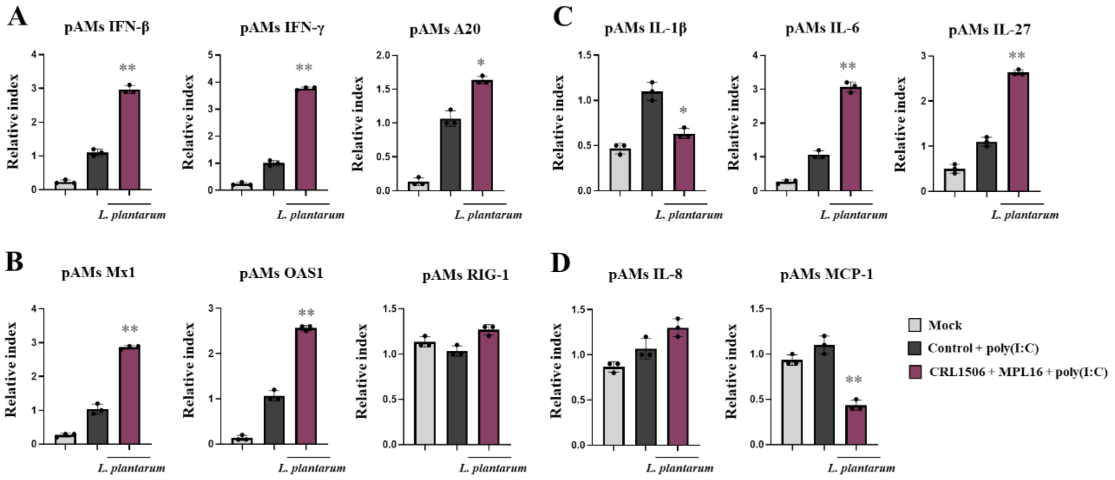

Figure S6. The effect of combined administration of *Lactiplantibacillus plantarum* CRL1506 and MPL16 on the cytokine profile of porcine alveolar macrophages induced by TLR3 activation. Porcine alveolar macrophages (pAMs) were stimulated with CRL1506+MPL16 strains for 24 hours and then stimulated with poly(I:C) for 12 hours. The expressions of interferons, antiviral factors, the regulatory factor A20, and proinflammatory and regulatory cytokines genes were determined. pAMs not treated with lactobacilli and stimulated with poly(I:C) were used as controls. The expressions of immune factors in pAMs without any stimuli (mock) are shown. The results are shown as mean ± SD. Significant differences are shown compared to the poly(I:C)-infected control group at $p < 0.05$ (*) or $p < 0.01$ (**).
